# Psychometric evaluation of the Inventory of Statements About Self-Injury (ISAS-I) in adolescents: evidence from SEM, Bayesian reliability, and item response theory

**DOI:** 10.3389/fpsyg.2026.1879759

**Published:** 2026-07-16

**Authors:** Jonatan Baños-Chaparro, Paul Ynquillay-Lima, Tomás Caycho-Rodríguez, Delly Santos-Chuquispuma, Renzo Santos-Chuquispuma

**Affiliations:** 1 Programa Académico de Psicología, Facultad de Ciencias de la Salud, Universidad Privada Norbert Wiener, Lima, Peru; 2 Instituto de Investigación, Universidad para el Desarrollo Andino, Huancavelica, Peru; 3 Facultad de Psicología, Universidad Científica del Sur, Lima, Peru

**Keywords:** adolescents, item response theory, mental health, non-suicidal self-injury, reliability

## Abstract

**Introduction:**

Non-suicidal self-injury constitutes a significant mental health issue among adolescents due to its association with depressive symptoms, anxiety, and an increased risk of suicidal behavior. Proper assessment of this behavior is essential for both research and clinical practice; however, in the Peruvian context, there are still limited instruments with solid evidence of validity and reliability.

**Objective:**

To analyze the psychometric properties of the Inventory of Statements About Self-Injury (ISAS-I) in Peruvian adolescents.

**Methods:**

Psychometric, cross-sectional, basic research with a quantitative approach. A total of 1,249 adolescents (56% male) participated and completed a sociodemographic questionnaire and three psychological instruments. The existing Spanish adaptation of the ISAS-I was used, and its content validity and comprehensibility were examined in the target population. Statistical analyses were conducted using structural equation modeling and item response theory.

**Results:**

The ISAS-I demonstrated adequate content validity (*V* > 0.70), a unidimensional structure (CFI = 0.97, RMSEA = 0.04 [90% CI: 0.041, 0.053], SRMR = 0.04), and adequate reliability (*ω* = 0.84, *H* = 0.90, *r_xx_* = 0.76). In addition, measurement invariance across sex was found (ΔCFI < 0.010 and ΔSRMR < 0.030), along with statistically significant associations with depressive symptoms and generalized anxiety. Item 7 showed the greatest discrimination and information capacity, and the scale demonstrated particular precision at higher levels of non-suicidal self-injury.

**Conclusion:**

The ISAS-I presents adequate evidence of validity and reliability in Peruvian adolescents, supporting its use as a brief and precise measure for assessing non-suicidal self-injury. These findings should be understood as psychometric evidence for the existing Spanish adaptation of the ISAS-I in this population, rather than as evidence of a new cross-cultural adaptation. Furthermore, its invariance across sex and its associations with mental health indicators support its utility in both clinical and research settings, promoting early detection and the design of preventive strategies in adolescent populations.

## Introduction

Non-suicidal self-injury (NSSI) is defined as deliberate and intentional harm to one’s own body without suicidal intent and constitutes a growing public health problem, particularly during adolescence, a stage characterized by intense emotional, cognitive, and social changes (Lurigio et al., 2024). According to a meta-analysis, the global lifetime and 12-month prevalence of NSSI among adolescents ranges between 22 and 23.2%, respectively (Xiao et al., 2022a,b). Another meta-analysis reports differential behavior by sex, with a higher frequency among females (21.4%) compared to males (13.7%), making it one of the most recurrent risk behaviors in this population (Moloney et al., 2024). In the Americas, two systematic reviews and meta-analyses indicate that reported rates also show a high lifetime prevalence, ranging between 11.5 and 20%, depending on the sociocultural context and assessment methods used (Lucena et al., 2022; Mannekote Thippaiah et al., 2021). In the Peruvian context, recent studies have shown a concerning trend, especially among school adolescents, where prevalence rates range from 19.8 to 47.4%, accompanied by emotional difficulties, generalized anxiety, and school bullying (Ancajima Carrasco and Cortez Vidal, 2022; Baños-Chaparro, 2023; Cabrera De la Cruz, 2021). This scenario highlights the need to further evaluate and understand NSSI in Peruvian adolescents through psychometrically sound instruments.

Mental health during adolescence represents a fundamental component of well-being and integral development, as multiple psychological and emotional disorders emerge during this stage and may persist into adulthood (World Health Organization, 2025). Among the main mental health problems in adolescents are child maltreatment, depression, generalized anxiety, behavioral disorders, substance use, and suicidal behaviors (Baños-Chaparro and Ynquillay-Lima, 2023; Baños-Chaparro et al., 2024; World Health Organization, 2025). In particular, NSSI has shown a close relationship with depressive symptoms and generalized anxiety, as many adolescents use self-injury as a maladaptive emotional regulation strategy (Wang et al., 2022; Wang et al., 2025). From the affect regulation model, NSSI temporarily reduces intense emotions such as sadness, guilt, distress, or emotional emptiness (Gray et al., 2022). Likewise, interpersonal theory suggests that self-injury may function as a way of communicating psychological suffering when adolescents perceive difficulties in verbally expressing their emotions or receiving social support (Robinson et al., 2024). On the other hand, cognitive-behavioral models propose that NSSI may be maintained through negative reinforcement processes, since the immediate relief of emotional distress increases the likelihood of repeating the behavior (Gray et al., 2022; Tilton-Weaver et al., 2022).

Scientific evidence has also identified important differences in the manifestation of non-suicidal self-injury according to sex. In general, adolescent females report higher rates of NSSI compared to males, especially in behaviors related to superficial cutting, accompanied by higher levels of depression, anxiety, self-criticism, and emotional dysregulation (Moloney et al., 2024; Xiao et al., 2022a,b). In contrast, adolescent males tend to present more externalizing behaviors, difficulties in impulse control, and lower emotional support-seeking (Moloney et al., 2024; Wang et al., 2023). From a theoretical perspective, these differences may be explained by gender socialization processes, which influence the way men and women express and regulate their emotions (Vafaei et al., 2023; Wang et al., 2023). Females tend to internalize emotional distress, increasing the risk of anxiety and depressive symptoms associated with NSSI, whereas males tend to externalize psychological distress through impulsive or aggressive behaviors (Moloney et al., 2024). In addition, sociocultural standards related to emotional expression may contribute to adolescent females resorting more frequently to self-injury as an emotional coping mechanism (Vafaei et al., 2023).

The proper assessment of non-suicidal self-injury in adolescents is essential for early detection, prevention, and clinical intervention. In this context, several psychological instruments have been developed to assess the frequency, characteristics, and functions of self-injury, including the Functional Assessment of Self-Mutilation (FASM; Lloyd et al., 1997), the Deliberate Self-Harm Inventory (DSHI; Gratz, 2001), the Self-Harm Behavior Questionnaire (SHBQ; Gutierrez et al., 2001), and the Inventory of Statements About Self-Injury (ISAS; Klonsky and Glenn, 2009). The latter, developed by Klonsky and Glenn (2009), has received special attention due to its brevity, ease of administration, clear interpretation, and adequate psychometric evidence across different cultural contexts. According to the literature, the ISAS-I was initially developed by Klonsky and Olino (2008) to specifically assess various NSSI behaviors. Subsequently, based on scientific evidence, the psychological functions of NSSI were incorporated, leading to the development of the ISAS-II, which integrated both components into the construction of the ISAS (Klonsky and Glenn, 2009). In particular, the ISAS-I is relevant because it allows the precise identification of the presence and frequency of different forms of self-injury, constituting a useful tool in both research and clinical and educational settings (Klonsky and Olino, 2008). Psychometric studies of the ISAS-I are extensive, with research conducted in Chile, China, Spain, and Türkiye confirming the structure and psychometric properties of the instrument across different cultural contexts (Bildik et al., 2013; Castro and Kirchner, 2017; Pérez et al., 2021; Tian et al., 2025). However, despite the increase in research on adolescent mental health in Peru, there is still limited evidence regarding instruments specifically validated to assess NSSI in Peruvian adolescents, highlighting the need to examine the psychometric performance, content validity, and comprehensibility of existing Spanish-language instruments in this population.

In this context, the present study is relevant due to the growing need for valid and reliable instruments to assess non-suicidal self-injury in Peruvian adolescents. The availability of psychometrically robust measures will strengthen processes of early detection, clinical assessment, and research in adolescent mental health (Lucena et al., 2022). Therefore, the main objective of the study is to analyze the psychometric properties of the ISAS-I in Peruvian adolescents. Specifically, the study aims to evaluate content validity through expert judgment; examine the comprehensibility of the existing Spanish adaptation in the target population through pilot administration; examine the factorial structure of the instrument using structural equation models; estimate reliability through classical and Bayesian approaches; analyze measurement invariance according to sex; explore the relationship between NSSI and other psychological variables such as depressive symptoms and generalized anxiety; and evaluate item functioning through Item Response Theory models. These analyses will allow the determination of the precision, stability, and usefulness of the ISAS-I in Peruvian adolescents, contributing evidence on the use of the existing Spanish adaptation in this population.

## Materials and methods

### Design

The study is framed within an instrumental design, as its purpose is to evaluate the psychometric properties of the ISAS-I (Ato et al., 2013). Likewise, it corresponds to a basic, cross-sectional study with a quantitative approach, aimed at determining the validity and reliability of the instrument in a sample of Peruvian adolescents.

### Participants

A total of 1,249 adolescents participated in the study, belonging to nine full-time public educational institutions affiliated with the Local Educational Management Unit (UGEL) of Angaraes. Participants were selected through non-probability convenience sampling and met the following inclusion criteria: (a) being enrolled and attending the selected educational institution, (b) being a secondary school student, (c) being between 10 and 19 years old, (d) providing informed consent from parents or guardians, and (e) providing informed assent from the adolescent. The age range established for adolescence was defined according to the World Health Organization classification (World Health Organization, 2017). The exclusion criteria were as follows: (a) presenting a diagnosis of intellectual or neurological developmental disorder that hindered completion of the survey, (b) not attending the institution during the data collection phase, (c) providing incomplete responses in the sociodemographic section or psychological instruments, and (d) voluntarily withdrawing from the study. The sample size estimation was conducted based on the hypothetical factorial model of the ISAS-I (12 items and one factor), considering four parameters: an expected CFI of 0.95, an average factor loading of 0.50, a statistical power of 99%, and a significance level of *p* = 0.05 (Arifin, 2026). Based on these criteria, a minimum required sample size of 653 participants was determined. The final sample exceeded this recommendation.

Regarding the distribution by school grade, the highest participation corresponded to fifth grade students (24.2%), followed by second grade (19.8%) and third grade (19.7%). The mean age was 15 years (SD = 1.49), ranging from 12 to 18 years, with participation from females (44%) and males (56%). Concerning participant characteristics, 82.1% reported not currently working, 73.2% indicated not being in a romantic relationship, and 2.2% reported being pregnant. Furthermore, when asked about their parents’ current marital status, 80% mentioned that both parents live together, while 12.7% indicated that their parents were separated. A small group of participants reported that their mother was widowed (3.4%) or that they had never known their father (2.5%), while others indicated that their father was widowed (1%), that they had never known their mother (0.2%), and some stated that they had never known either their father or mother (0.2%).

### Measures

#### Inventory of Statements About Self-Injury (ISAS)

The ISAS is a self-report instrument that assesses non-suicidal self-injury, divided into two sections: non-suicidal self-injury behaviors (ISAS-I), consisting of 12 items, and functions of non-suicidal self-injury (ISAS-II), consisting of 39 items. The present study used the existing Spanish adaptation of the ISAS-I by Pérez et al. (2021). No new forward translation, back translation, expert reconciliation, or formal cross-cultural adaptation procedure was conducted in the present study. Instead, the Spanish version was reviewed to evaluate content validity and comprehensibility in Peruvian adolescents. In this study, the first section (ISAS-I) was administered, which examines the lifetime frequency of intentional non-suicidal self-injury behaviors performed without suicidal intent. Following the ISAS-I administration format, participants were instructed to report only intentional self-injurious behaviors carried out without the intention of dying or ending their life. This instruction was presented as a general criterion for responding to all 12 NSSI methods, rather than as a separate intent assessment for each individual behavior. Specifically, each item includes five response categories (0 = no presence of any type of self-injury, 1 = between 1 and 4 times, 2 = between 5 and 50 times, 3 = between 51 and 100 times, 4 = more than 100 times). Accordingly, the psychometric evidence reported in this study applies to the Spanish adaptation of the ISAS-I with this five-category ordinal response format. Higher scores should be interpreted as reflecting greater lifetime frequency or overall behavioral involvement in NSSI, rather than as a direct indicator of clinical severity, medical lethality, psychological function, or equivalence among different self-injurious behaviors. In this study, the Spanish version was used, and its psychometric properties were examined in the current research (Pérez et al., 2021).

Content validity and comprehensibility were assessed by four expert judges, who were purposively selected according to the following pre-established criteria: (a) academic training in psychology and a doctoral degree, (b) more than 5 years of professional and/or research experience in mental health, and (c) familiarity with the evaluation of psychological instruments. A panel of four experts was considered adequate for this initial content review because methodological recommendations suggest the participation of at least three experts in content validity studies (Roebianto et al., 2023). Subsequently, comprehensibility was examined through a pilot administration involving seven adolescents who met the study eligibility criteria.

#### Patient Health Questionnaire-2 (PHQ-2)

The PHQ-2 is a brief two-item questionnaire that assesses depressive symptoms during the past 2 weeks. Each item is answered on a four-point scale ranging from 0 (not at all) to 3 (nearly every day). Total scores range from 0 to 6, with higher scores indicating greater depressive symptomatology. In the present study, the Peruvian adaptation of the instrument was used, which demonstrated adequate reliability levels (*ω* = 0.73) (Villarreal-Zegarra et al., 2023).

#### Generalized Anxiety Disorder-2 (GAD-2)

The GAD-2 is a brief scale composed of two items that assesses symptoms of generalized anxiety experienced during the past 2 weeks. Each item is rated on a four-point scale ranging from 0 (not at all) to 3 (nearly every day). Total scores range from 0 to 6, with higher scores reflecting greater levels of generalized anxiety. For this study, the version adapted to the Peruvian context was used, which showed adequate internal consistency (*ω* = 0.88) (Baños-Chaparro, 2022).

### Procedure

Data collection was carried out after obtaining formal authorization from nine educational institutions belonging to the Local Educational Management Unit (UGEL) of Angaraes, located in the district of Lircay, department of Huancavelica, Peru. Before fieldwork, authorization to access the schools and administer the questionnaires was obtained from the corresponding educational authorities [UGEL of Angaraes with registration number 245-2022]. Fieldwork was conducted in person during September and October 2022, following a schedule previously coordinated with the educational authorities of each participating institution.

Before administering the instruments, the researchers provided students with a detailed explanation of the objectives and scope of the study, emphasizing the academic and scientific nature of the research. Participants were also informed that participation was entirely voluntary and that their responses would be treated anonymously and confidentially, ensuring the protection of their identity and the exclusive use of the information for research purposes. Likewise, it was emphasized that students were free to withdraw from the study at any time without any academic or personal consequences.

The questionnaires were administered during school hours and in spaces provided by the educational institutions, ensuring adequate conditions to facilitate comprehension and the proper completion of the assessment. The application protocol began with the reading and acceptance of the informed assent by the adolescent participants, after obtaining informed consent from their parents. Subsequently, a sociodemographic form was completed to collect basic student information, and finally, the psychological instruments included in the research were administered, following a standardized order of application to ensure consistency in the data collection process. Because the ISAS-I was administered in a school-based group format, the distinction between NSSI and suicidal behavior was established through the general instruction of the instrument, which asked participants to report only self-injurious behaviors performed without suicidal intent. Suicidal intent was not assessed separately for each specific method. Therefore, suicide attempts or behaviors with undetermined intent were not clinically adjudicated during data collection, and responses should be understood as self-reported NSSI according to the instructions provided to participants.

### Statistical analysis

The statistical analysis was conducted rigorously, sequentially, and in a methodologically structured manner using RStudio software version 4.3.2, following a comprehensive approach aimed at the advanced psychometric evaluation of the instrument. This procedure included several complementary analytical stages, which allowed for an exhaustive examination of the metric properties of the ISAS-I.

In the first phase, a detailed descriptive analysis of each item was performed in order to identify its initial statistical behavior and assess its preliminary psychometric quality. To this end, measures of central tendency and dispersion were calculated, specifically the mean and standard deviation, as well as measures related to the distribution of responses, such as skewness and kurtosis coefficients. In addition, corrected item-total correlations were estimated, considering values greater than 0.30 as the minimum acceptable criterion, in accordance with the guidelines proposed by Kline (2023). Furthermore, content validity was examined using Aiken’s *V* coefficient, evaluating fundamental dimensions such as clarity, relevance, and representativeness of the items. Values greater than 0.70 were considered adequate, following methodological recommendations reported in recent studies (Roebianto et al., 2023).

In the second stage, a confirmatory factor analysis (CFA) was conducted to empirically verify the internal structure of the instrument and determine the degree of correspondence between the theoretical model and the observed data. A one-factor model was specified *a priori*, consistent with the original purpose of the ISAS-I as a measure of the frequency of different NSSI methods rather than distinct functional dimensions (Klonsky and Olino, 2008). Alternative factor structures were not tested because the ISAS-I does not propose theoretically defined behavioral subscales. Since the responses were ordinal in nature, the Weighted Least Squares Mean and Variance Adjusted (WLSMV) estimator was used, as it is widely recommended for this type of categorical variable. The CFA was estimated using the polychoric correlation matrix, which is appropriate for ordinal response categories. Model fit quality was assessed using several goodness-of-fit indices, including the chi-square statistic (*χ*^2^), degrees of freedom (d*f*), and *p*-value, as well as the Comparative Fit Index (CFI > 0.90), Tucker-Lewis Index (TLI > 0.90), the Root Mean Square Error of Approximation (RMSEA < 0.08), and the Standardized Root Mean Square Residual (SRMR < 0.08), as indicators of satisfactory fit (Jordan Muiños, 2021). At the same time, standardized factor loadings were analyzed, considering values above 0.30 acceptable, as they reflect an adequate contribution of each item to the construct being assessed (Kline, 2023).

Subsequently, in a third phase, the reliability of the instrument was evaluated using the Bayesian omega coefficient (*ω*), coefficient *H*, and empirical reliability (*r_xx_*). The use of these indicators allowed the limitations associated with traditional reliability estimation methods to be overcome, providing a more stable and realistic assessment of the precision of the obtained scores. This approach is supported by recent studies recommending the use of modern metrics for advanced psychometric research (Baños-Chaparro and Caycho-Rodríguez, 2024; Gunnell, 2020; Pfadt et al., 2022). In this study, the prior distribution of the Bayesian omega was set at *r* = 0.707, in accordance with simulation studies (Pfadt et al., 2023). In addition, model convergence was evaluated using the *R*-hat index, with values close to 1 indicating adequate convergence of the Markov chains, based on 1,000 iterations (Pfadt et al., 2022).

In the fourth stage, factorial invariance according to sex was examined in order to determine whether the instrument measured the construct equivalently in males and females. Initially, a configural model was tested to verify the similarity of the basic factorial structure across both groups. Subsequently, progressive constraints were introduced to evaluate different levels of metric equivalence: threshold invariance, metric invariance, scalar invariance, and strict invariance. Comparisons between models were conducted by evaluating minimal changes in fit indices, using ΔCFI < 0.010 and ΔSRMR < 0.030 as acceptance criteria (Chen, 2007; Finch and French, 2018).

In a fifth phase, a covariance-based structural equation modeling (CB-SEM) approach was implemented to analyze the structural relationships among the latent variables included in the study. For this purpose, the robust maximum likelihood estimator (MLR) was used, together with fit indices such as CFI, RMSEA, and SRMR, maintaining the same previously established acceptability criteria (Jordan Muiños, 2021). Additionally, the magnitude of the observed relationships was interpreted using the cutoff points suggested by Cohen (1988), considering effect sizes as small = 0.10, moderate = 0.30, and large = 0.50.

Finally, in the sixth stage, an Item Response Theory (IRT) analysis was conducted using a two-parameter Graded Response Model (GRM), which can be understood as a polytomous extension of the 2PL model for ordered response categories. This specification was selected because the ISAS-I items have ordinal polytomous response categories. The model was estimated using the “mirt” package, version 1.44.0, through full-information item factor analysis with one latent factor. Parameter estimation was performed using the EM algorithm, with BFGS as the M-step optimizer and Ramsay acceleration. The model converged within a tolerance criterion of 1*e* − 04 after 60 EM iterations, with a final maximum change of 0.00008. The information matrix was estimated using the Oakes method, with 61 rectangular quadrature points and a Gaussian latent density. The model estimated the discrimination parameter (*a*), which evaluates the ability of items to differentiate between individuals with different levels of the latent trait (*θ*), considering values above 1 as adequate (Baker and Kim, 2017). Likewise, the category threshold parameters (*β*) were estimated, representing the points on the latent trait continuum at which respondents have a greater probability of endorsing a given response category or a higher category. Additionally, key model assumptions were verified, such as unidimensionality, local independence through the inspection of standardized residual correlations derived from the LD residual matrix in mirt, where the upper triangle represents signed Cramer’s *V* coefficients. Values closer to zero were interpreted as evidence of no relevant local dependence among item pairs, and monotonicity, examined using a nonparametric Mokken model with the critical statistic (crit < 0.40) (Sijtsma and van der Ark, 2017; Stover et al., 2019).

The precision and informative capacity of the model were analyzed through the Item Information Curves (IIC) and the Test Information Curve (TIC), allowing identification of the levels of the latent trait at which the scale demonstrated greater measurement accuracy. To ensure proper model specification, global fit was examined using the *C*^2^ statistic, especially recommended for IRT models applied to ordinal data, complemented with fit indices such as CFI > 0.90, RMSEA2 < 0.089, and SRMR < 0.05 (Cai et al., 2023; Lubbe and Schuster, 2019; Maydeu-Olivares and Joe, 2014; Monroe and Cai, 2015). Finally, local item fit was evaluated using the *S* − *X*^2^ index, the RMSEA. *S* − *X*^2^ criterion below 0.06 (Bean and Bowen, 2021; Kang and Chen, 2008), and infit and outfit statistics, considering values between 0.50 and 1.5 as evidence of optimal item functioning within the model (Wright and Linacre, 1994).

### Ethical considerations

The study adhered to the ethical guidelines of the American Psychological Association and the Code of Ethics of the Colegio de Psicólogos del Perú, as described in Chapter Six on good research practices (Colegio de Psicólogos del Peru, 2024; Knapp and VandeCreek, 2003). The chronology of ethical and institutional authorization was as follows. Data collection was conducted in September and October 2022 after obtaining formal authorization from the participating educational institutions and/or the corresponding educational authority [UGEL of Angaraes with registration number 245-2022]. Before participation, parents or guardians provided informed consent, and adolescents provided informed assent. The survey was anonymous and voluntary, and data confidentiality was guaranteed. Subsequently, the current psychometric study using the anonymized dataset was reviewed and approved by the Ethics Committee of the Universidad para el Desarrollo Andino under registration number II-UDEA-2024-001. Therefore, the 2024 ethics review should be understood as a subsequent review of the anonymized dataset and the present psychometric analysis, not as an approval that preceded the 2022 school-based data collection.

Given that the study involved minors and direct questions about self-injury, a risk management procedure was established during school-based administration. Before completing the questionnaires, participants were informed that they could stop answering at any time or request assistance if any item caused discomfort. The research team remained present during administration to monitor signs of distress and to provide immediate support if needed. Although questionnaire responses were anonymous and were not used for individual clinical screening, anonymity did not prevent intervention in the event of an imminent risk disclosed directly by a participant or observed during administration. In such cases, the planned procedure was to interrupt participation, provide immediate containment, and notify the school authority or school psychologist/tutor responsible for student welfare, in coordination with the parents or guardians when appropriate. At the end of the administration, students were reminded that they could seek support from school counseling/tutoring services or local mental health services. During data collection, no participant requested assistance, showed acute distress, or disclosed imminent self-harm or suicide risk requiring activation of the referral protocol.

## Results

### Evidence based on content

Expert judges conducted a systematic evaluation of the item content, considering its relevance, degree of construct representativeness, and clarity of wording. This procedure was intended to provide evidence of content validity of the existing Spanish adaptation in the Peruvian adolescent context, rather than evidence of a complete cross-cultural adaptation process. As a result, Aiken’s *V* coefficients exceeded the cutoff point of 0.70 in all cases (see Table 1), supporting the adequacy of the content. Consequently, all participating experts (*n* = 4) approved the final version of the ISAS-I without requiring additional modifications. Consistently, during the pilot administration (*n* = 7), participants did not report comprehension difficulties or suggest changes, indicating that the items were clear and appropriate for the target population. Therefore, the evidence obtained at this stage should be interpreted as preliminary support for clarity, relevance, representativeness, and comprehensibility in the target population, given the limited size of both the expert panel and the pilot group.

**Table 1:** Content validity of the ISAS-I items.

Items	Relevance (*n* = 4)		Representativeness (*n* = 4)		Clarity (*n* = 4)	
	*V*	CI 95%	*V*	CI 95%	*V*	CI 95%
1	0.83	0.60, 0.94	0.83	0.60, 0.94	0.75	0.51, 0.90
2	0.92	0.70, 0.98	0.92	0.70, 0.98	0.92	0.70, 0.98
3	0.75	0.51, 0.90	0.83	0.60, 0.94	0.92	0.70, 0.98
4	0.92	0.70, 0.98	0.92	0.70, 0.92	0.92	0.70, 0.92
5	0.92	0.70, 0.98	0.83	0.60, 0.94	0.83	0.60, 0.94
6	0.75	0.51, 0.90	0.75	0.51, 0.90	0.75	0.51, 0.90
7	0.83	0.60, 0.94	0.83	0.60, 0.94	0.83	0.60, 0.94
8	0.83	0.60, 0.94	0.83	0.60, 0.94	0.83	0.60, 0.94
9	0.92	0.70, 0.98	0.92	0.70, 0.98	0.92	0.70, 0.98
10	0.92	0.70, 0.98	0.92	0.70, 0.98	0.83	0.60, 0.94
11	0.92	0.70, 0.98	0.92	0.70, 0.98	0.75	0.51, 0.90
12	0.83	0.60, 0.94	0.83	0.60, 0.94	0.83	0.60, 0.94

*V*, Aiken’s *V*; CI, confidence intervals.

### Descriptive analysis

Table 2 presents the descriptive statistics of the scale items. Overall, the means ranged from 0.15 (item 12) to 0.74 (item 6), suggesting a low frequency of the behaviors assessed in the sample. Regarding the standard deviation, the highest value was found in item 6 (SD = 1.01), whereas the lowest value was found in item 12 (SD = 0.50). Regarding the shape of the distributions, skewness coefficients were positive for all items (range = 1.57 to 4.25), indicating a right-skewed distribution. Likewise, kurtosis values ranged from 2.07 to 21.47, indicating leptokurtic distributions, especially in the less frequent items. Regarding item discrimination, correlations ranged between 0.40 and 0.60. Overall, these values suggest an adequate discriminative capacity of the items, although some showed more moderate indices (e.g., items 9, 11, and 12).

**Table 2:** Descriptive measures.

Item	*M*	SD	*g*_1_	*g*_2_	*r*_it_
1. Hitting oneself hard	0.65	0.97	1.75	2.85	0.56
2. Pulling out one’s hair	0.44	0.84	2.42	6.28	0.49
3. Pinching oneself	0.55	0.90	1.98	3.90	0.53
4. Cutting oneself	0.39	0.84	2.62	7.03	0.45
5. Biting oneself	0.37	0.77	2.64	7.69	0.57
6. Interfering with wound healing (e.g., picking at a scab)	0.74	1.01	1.57	2.07	0.52
7. Scratching oneself hard	0.51	0.87	2.07	4.37	0.60
8. Rubbing skin against rough surfaces	0.32	0.70	2.81	9.04	0.56
9. Burning oneself	0.28	0.64	2.80	8.75	0.41
10. Sticking needles into oneself	0.36	0.74	2.48	6.65	0.51
11. Scraping one’s skin	0.28	0.66	3.10	11.37	0.40
12. Ingesting harmful substances	0.15	0.50	4.25	21.47	0.44

*M*, media; SD, standard deviation; *g*_1_, skewness; *g*_2_, kurtosis; *r*_it_, corrected item test correlation.

### Evidence based on internal structure

**Figure 1:**
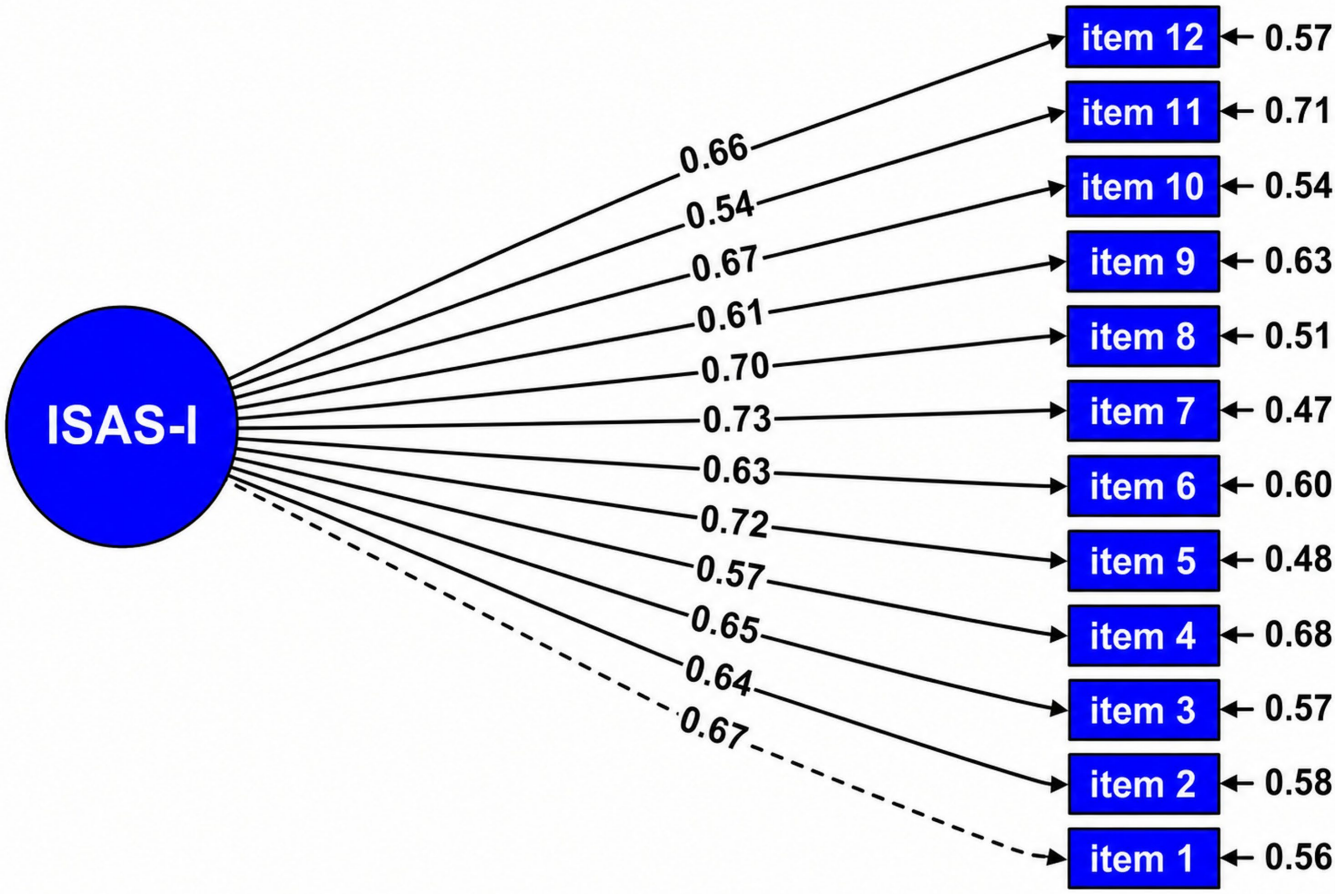
Factor structure of the ISAS-I.

The confirmatory factor analysis showed that the structure of the ISAS-I is adequately organized into a single factor, supporting its unidimensional nature. The fit indices demonstrated satisfactory model performance (*χ*^2^ = 226.01, d*f* = 54, *p* = 0.001, CFI = 0.97, TLI = 0.96, RMSEA = 0.047 [90% CI: 0.041, 0.053], SRMR = 0.04). Likewise, the standardized factor loadings ranged from 0.54 to 0.73, indicating that all items consistently contribute to the measurement of the assessed phenomenon (Figure 1).

### Reliability

The Bayesian omega coefficient showed adequate reliability (*ω* = 0.84 [95% CI: 0.83, 0.85]), suggesting that the items maintain a homogeneous relationship and consistently measure the underlying construct. In addition, it showed adequate convergence, with Rhat = 1.000. Similarly, coefficient *H* = 0.90 and *r_xx_* = 0.76 showed acceptable values, which reinforces the psychometric quality of the scale scores.

### Measurement invariance

Table 3 presents the results of the factorial measurement invariance analysis of the ISAS-I by sex. The models were tested sequentially, beginning with the configural model and progressively imposing equality constraints on thresholds, factor loadings, intercepts, and residuals. The more restrictive models did not show a meaningful deterioration in fit when compared with the previous models, as the changes in the fit indices remained within the recommended criteria (ΔCFI < 0.010 and ΔSRMR < 0.030). These findings support the measurement invariance of the ISAS-I across females and males, suggesting that the instrument assesses the construct equivalently in both groups and permits meaningful comparisons between sexes. However, these results should not be interpreted as evidence that observed sex differences are necessarily “true” differences, but rather as evidence that comparisons between groups are psychometrically appropriate.

**Table 3:** Invariance analysis of the ISAS-I by sex.

Models	*X*^2^ (gL)	*p*	CFI	SRMR	∆CFI	∆SRMR
M1	290.09 (108)	0.001	0.970	0.051	—	—
M2	323.92 (132)	0.001	0.968	0.051	0.002	0.000
M3	311.96 (143)	0.001	0.972	0.052	0.004	0.001
M4	324.84 (154)	0.001	0.972	0.055	0.000	0.003
M5	387.27 (166)	0.001	0.963	0.055	0.009	0.000

M1, configural invariance; M2, threshold invariance; M3, metric invariance; M4, scalar invariance; M5, strict invariance.

### Evidence based on the relationship with other variables

**Figure 2:**
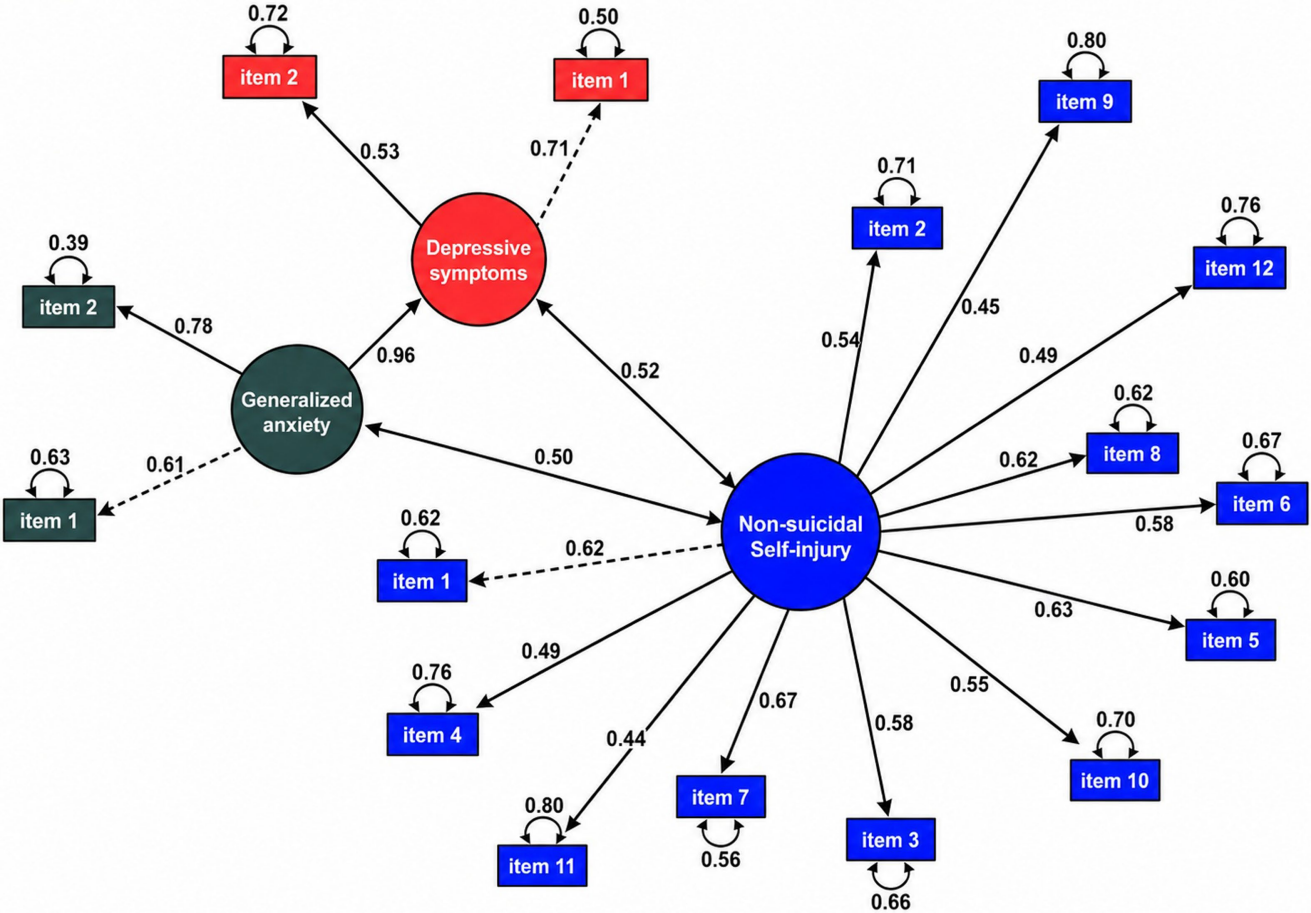
Structural model of the relationship between non-suicidal self-injury, generalized anxiety, and depressive symptoms in adolescents. Solid lines indicate the trajectories of standardized correlations between latent variables and the factor loadings of observed indicators, while dashed lines represent measurement error variances.

The convergent model among non-suicidal self-injury, generalized anxiety, and depressive symptoms is presented in Figure 2. In particular, the structural model showed an adequate fit to the empirical data (CFI = 0.97, RMSEA = 0.03 [90% CI: 0.023, 0.031], SRMR = 0.03). Non-suicidal self-injury was found to be positively and statistically significantly associated, with a large effect size, with generalized anxiety (*r* = 0.50, *p* = 0.001) and depressive symptoms (*r* = 0.52, *p* = 0.001).

### Item response theory

**Figure 3:**
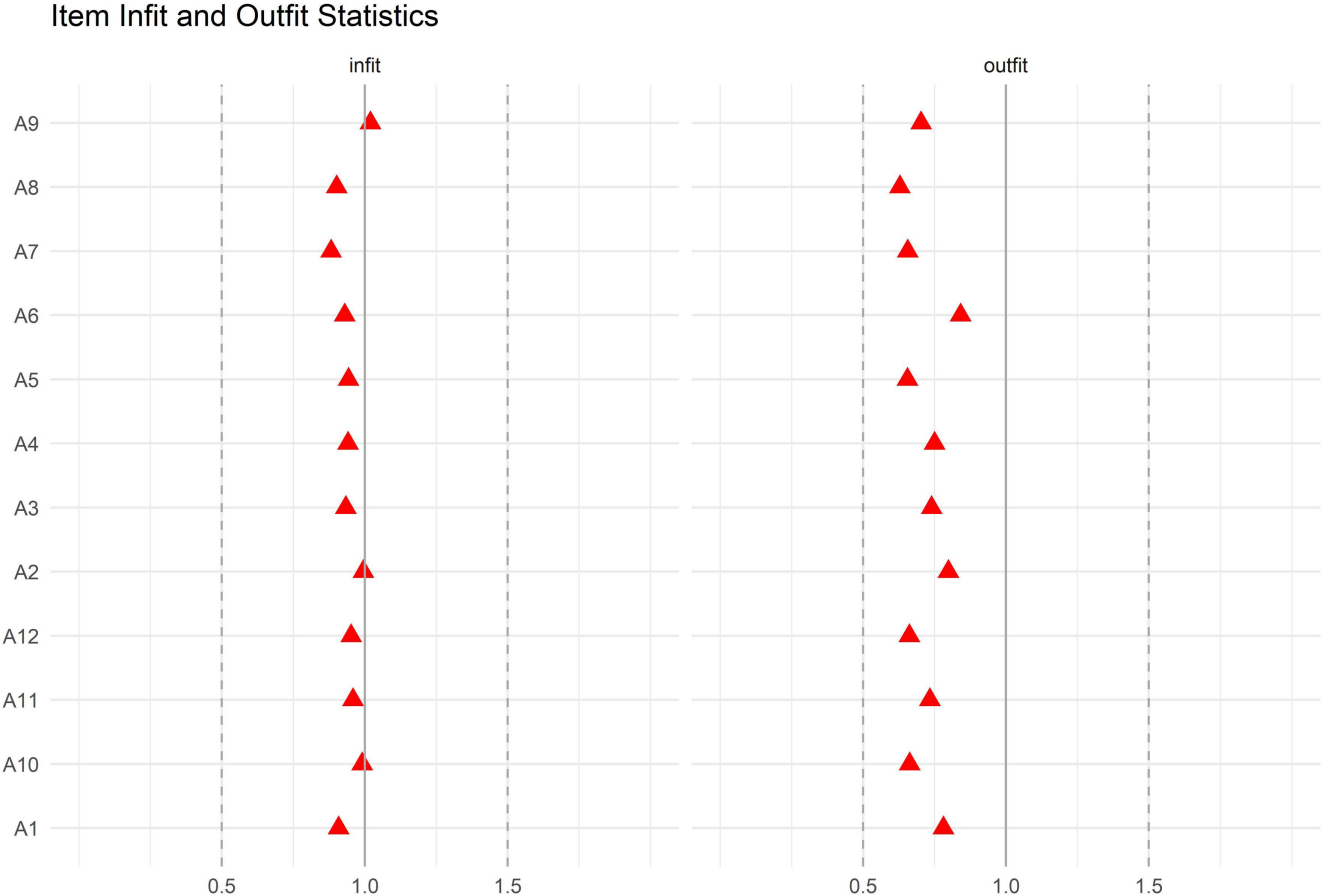
Item Infit and Outfit Statistics of the ISAS-I.

The fundamental assumptions of the model were carefully analyzed before proceeding with the interpretation of the results. The evidence obtained through confirmatory factor analysis supported the existence of a unidimensional structure, while local independence was corroborated through the LD-*X*^2^ index, whose standardized association values remained at low levels (between −0.097 and 0.131). Likewise, monotonicity did not detect any relevant violations (crit < 0.40). Similarly, the infit and outfit statistics remained within the acceptable criteria of ±1.5, confirming the adequate functioning of the items (Figure 3).

Overall, the model showed a satisfactory fit to the empirical data, reflected in robust goodness-of-fit indicators (*C*^2^ = 177.24, d*f* = 54, RMSEA2 = 0.03 [90% CI: 0.033, 0.046], CFI = 0.99, and SRMR = 0.05). These results indicate that the proposed structure adequately reproduces the observed relationships in the data. Complementarily, the item-fit analysis reported in Table 4 showed optimal values (RMSEA·*S* − *X*^2^ < 0.06), further supporting the model fit.

**Table 4:** Discrimination, difficulty, and item fit parameters.

Items	Item parameters					Item fit	
	*a*	*β*_1_	*β*_2_	*β*_3_	*β*_4_	*S* − *x*^2^ (gL)	RMSEA·*S* − *x*^2^
1	1.54	0.322	1.535	2.415	2.905	107.924 (69)	0.020
2	1.51	0.830	2.026	2.848	3.147	59.308 (56)	0.006
3	1.55	0.523	1.747	2.530	3.119	108.312 (70)	0.019
4	1.17	1.199	2.413	3.222	3.785	70.117 (71)	0.001
5	1.84	0.925	1.953	2.707	3.083	52.210 (54)	0.001
6	1.43	0.102	1.522	2.278	2.975	99.727 (72)	0.016
7	1.88	0.543	1.662	2.357	2.898	76.639 (62)	0.013
8	1.72	1.045	2.200	2.946	3.525	73.201 (52)	0.017
9	1.39	1.328	2.529	3.518	4.529	80.570 (52)	0.019
10	1.63	0.986	2.094	2.902	3.704	96.801 (56)	0.022
11	1.09	1.545	3.094	4.193	4.740	63.959 (53)	0.012
12	1.58	1.848	2.833	3.742	4.304	60.316 (42)	0.017

*a*, discrimination parameter; *β*, difficulty parameter; *S* − *x*^2^, fit index; d*f*, degrees of freedom; RMSEA·*S* − *x*^2^, root mean square error of approximation.

**Figure 4:**
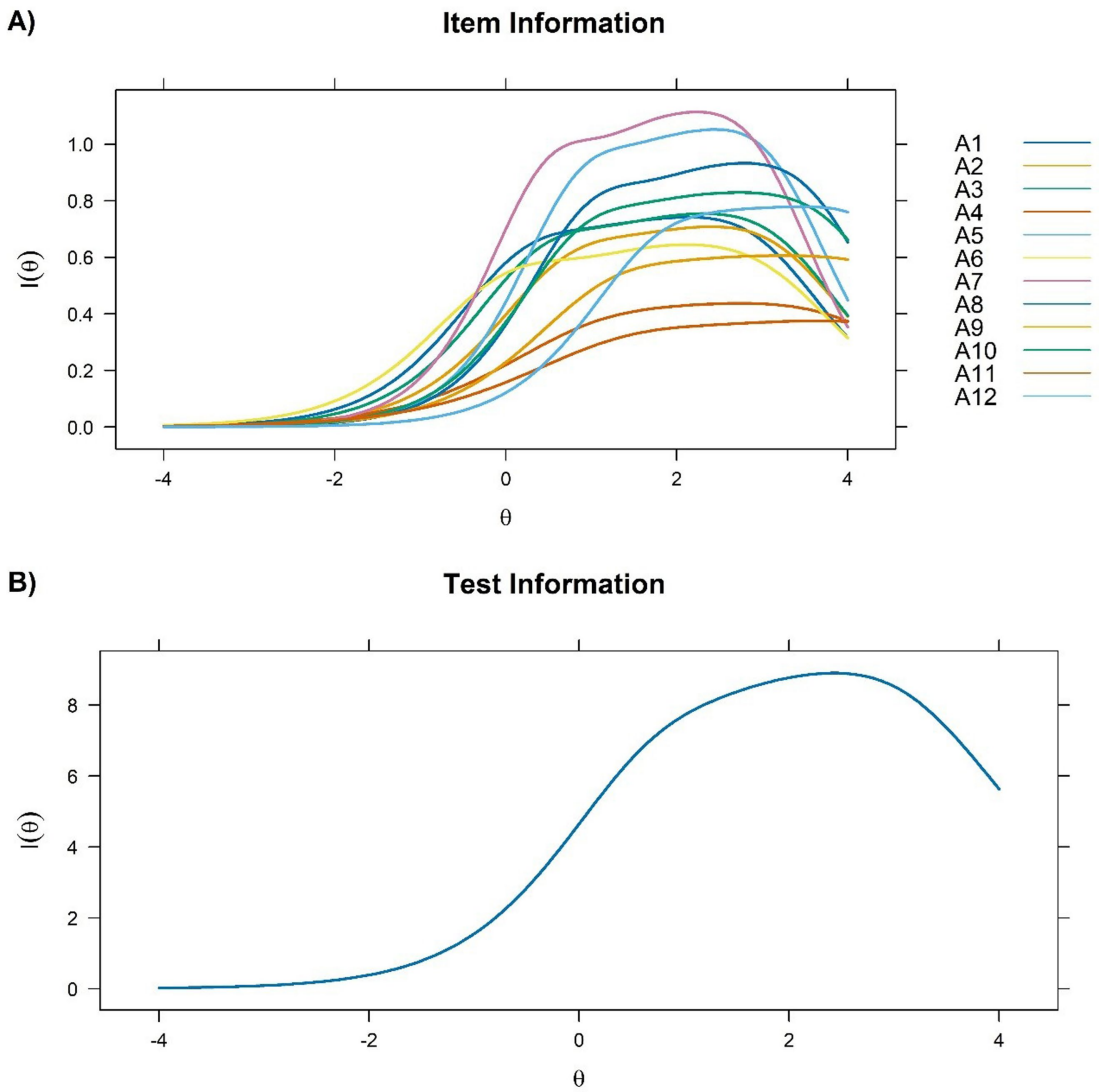
Item information function [Section **(A)**] and test information function [Section **(B)**] of the ISAS-I.

Regarding the estimated parameters, item 7 stood out by presenting the highest discrimination index (*a*), indicating a high capacity to differentiate between individuals with different levels of the evaluated latent trait. In the present study, this latent trait was conceptualized as a general propensity toward, or overall involvement in, lifetime NSSI behaviors, as reflected by the frequency of heterogeneous self-injurious acts. It should not be interpreted as a direct measure of clinical severity or medical risk. Concerning the difficulty parameter (*β*), a progressive trend was observed in the selection of higher response categories as *θ* levels increased, demonstrating coherent functioning of the response options. Finally, Figure 4 showed that item 7 contributed the greatest amount of information within the scale. The test information function suggests that the ISAS-I measures more precisely at higher levels of NSSI behavioral involvement, without implying diagnostic accuracy, clinical cutoff validity, or prediction of suicide risk.

## Discussion

Non-suicidal self-injury (NSSI) currently constitutes one of the main mental health problems among adolescents due to its high prevalence and association with multiple indicators of psychological vulnerability, such as depressive symptoms, generalized anxiety, emotional regulation difficulties, and suicidal behaviors (Lurigio et al., 2024; Wang et al., 2022; World Health Organization, 2025). In this context, the availability of valid and reliable instruments to assess NSSI is essential for early detection, research, and the planning of preventive interventions in adolescent populations. However, in the Peruvian context, there is still limited psychometric evidence regarding specific measures for assessing NSSI, particularly among school adolescents. Therefore, the aim of the present study was to analyze the psychometric properties of the ISAS-I in Peruvian adolescents through a comprehensive approach incorporating SEM, Bayesian reliability, measurement invariance analysis, and Item Response Theory (IRT).

Regarding the internal structure, the results showed that the ISAS-I presents a unidimensional factorial structure in Peruvian adolescents. This finding is consistent with previous research conducted in different cultural contexts, including Turkey (Bildik et al., 2013), Spain (Pérez et al., 2021), Chile (Castro and Kirchner, 2017), the United States (Klonsky and Olino, 2008), and China (Tian et al., 2025), where it was also reported that the different NSSI behaviors can be understood as observable manifestations of a general latent construct related to NSSI. This result should be interpreted as reflecting a general level of lifetime NSSI behavioral involvement across different methods, rather than the clinical equivalence of those behaviors or a single common psychological mechanism (Klonsky and Olino, 2008). The psychological functions of NSSI are assessed separately in the ISAS-II; therefore, the unidimensional model pertains specifically to the behavioral section of the instrument (Klonsky and Glenn, 2009). From a theoretical perspective, this unidimensional structure suggests that the different forms of self-injury share common underlying psychological mechanisms, especially those associated with emotional regulation, relief from psychological distress, and maladaptive coping (Gray et al., 2022). However, the presence of a unidimensional structure should not be taken to mean that all ISAS-I behaviors are clinically equivalent or that the total score directly represents clinical severity. The 12 behaviors differ in medical risk, frequency, psychological meaning, and possible overlap with other clinical conditions. Therefore, the latent factor identified in this study is more cautiously interpreted as a general dimension of NSSI behavioral involvement or frequency burden, rather than as a direct severity continuum. This interpretation supports the use of the ISAS-I as a brief screening tool, while also emphasizing the need to consider item-level responses in clinical assessment.

Regarding reliability, the results showed adequate levels of internal consistency through the Bayesian omega coefficient, coefficient H, and the *r_xx_* coefficient derived from IRT. These findings are consistent with previous studies conducted in other European and Asian countries, where the ISAS-I demonstrated adequate levels of precision in measuring NSSI (Bildik et al., 2013; Castro and Kirchner, 2017; Klonsky and Glenn, 2009; Pérez et al., 2021; Tian et al., 2025). However, the present study significantly expands the available psychometric evidence by incorporating Bayesian reliability estimates, an aspect that had not previously been reported in studies on the ISAS-I. Bayesian reliability represents a methodologically robust alternative to classical approaches because it allows for more stable estimates through posterior distributions and the incorporation of parameter uncertainty (Pfadt et al., 2022). In addition, the combined use of coefficient *H* and *r_xx_* strengthens the precision of psychometric inferences because it integrates evidence from both classical test theory and probabilistic IRT models (Baker and Kim, 2017). Overall, these results suggest that the ISAS-I provides consistent and precise scores for assessing NSSI in Peruvian adolescents.

Regarding measurement invariance, the results indicated that the ISAS-I demonstrates equivalence across sex, showing that the instrument assesses the NSSI construct comparably between males and females. This finding is particularly relevant because previous studies on the ISAS-I had not reported measurement invariance analyses (Bildik et al., 2013; Castro and Kirchner, 2017; Klonsky and Glenn, 2009; Pérez et al., 2021; Tian et al., 2025). Consequently, the present study constitutes one of the first empirical contributions demonstrating evidence of structural equivalence of the ISAS-I according to sex in adolescents. From a psychometric perspective, invariance ensures that observed differences between males and females reflect true differences in NSSI rather than biases derived from differential item functioning (Chen, 2007; Finch and French, 2018). This finding is especially relevant considering that the literature reports important differences in the prevalence and expression of self-injury according to sex, where females tend to report higher levels of NSSI associated with internalizing symptoms, while males present more externalizing behaviors and impulsivity (Moloney et al., 2024; Wang et al., 2023). Therefore, the evidence of invariance allows for valid and reliable comparisons between both groups.

Likewise, evidence of validity based on the relationship with other variables was found, observing significant associations between NSSI, depressive symptoms, and generalized anxiety. These results are consistent with the scientific literature indicating that adolescents with higher levels of depression and anxiety are more likely to engage in self-injurious behaviors (Wang et al., 2022; Wang et al., 2025). One possible explanation for these associations is that self-injury functions as a maladaptive emotional regulation strategy in response to intense emotions, negative thoughts, and persistent states of psychological distress (Gray et al., 2022). From cognitive-behavioral models, NSSI may be maintained through negative reinforcement, since temporary relief from emotional distress increases the likelihood of repeating the behavior (Tilton-Weaver et al., 2022). Likewise, depressive symptoms are often associated with feelings of emptiness, self-criticism, and hopelessness, whereas generalized anxiety involves high levels of emotional tension and persistent worry, factors that may increase vulnerability to self-injurious behaviors as a mechanism for emotional relief (Wang et al., 2025). Consequently, the obtained results support the convergent validity of the ISAS-I and reinforce the multidimensional understanding of NSSI in adolescents.

Regarding the IRT analyses, item 7 (“Scratching oneself hard”) presented the highest levels of discrimination and information within the scale. This finding suggests that this item has a high capacity to differentiate adolescents with different levels of the latent NSSI trait, especially at high levels of severity (Xiao et al., 2022a,b; Moloney et al., 2024). One possible explanation may be related to the fact that this behavior represents a relatively frequent, observable, and culturally recognizable manifestation among adolescent self-injurious behaviors, facilitating more consistent and differentiated responses (Lurigio et al., 2024). In addition, the results showed that the ISAS-I demonstrates greater precision at high levels of NSSI, which is clinically relevant because the instrument is especially sensitive for identifying adolescents at greater psychological risk (Klonsky and Glenn, 2009). From the IRT perspective, this implies that the scale provides greater psychometric information in individuals with high construct severity, favoring screening processes and the identification of clinically relevant cases (Baker and Kim, 2017; Bean and Bowen, 2021). It is noteworthy that previous psychometric studies of the ISAS-I had not incorporated IRT-based analyses, focusing mainly on classical test theory approaches (Bildik et al., 2013; Castro and Kirchner, 2017; Klonsky and Glenn, 2009; Pérez et al., 2021; Tian et al., 2025).

At the theoretical level, the findings contribute to strengthening the dimensional conceptualization of NSSI in adolescents, supporting the idea that different self-injurious behaviors can be understood as observable expressions of the same psychological continuum associated with dysfunctional emotional regulation and maladaptive coping (Gray et al., 2022; Tilton-Weaver et al., 2022). Likewise, the evidence of validity, reliability, invariance, and IRT provides empirical support for the structural stability and precision of the ISAS-I, favoring a more comprehensive and accurate assessment of self-injurious behaviors in adolescent populations (Bean and Bowen, 2021; Stover et al., 2019). Similarly, the present study expands the international literature on the ISAS-I by incorporating contemporary psychometric approaches, such as Bayesian reliability, measurement invariance analysis, and item response theory, aspects scarcely reported in previous research on NSSI (Pfadt et al., 2022).

From a clinical and community perspective, the results suggest that the ISAS-I may constitute a useful tool for the early detection of self-injurious behaviors in Peruvian adolescents, particularly in school settings, community mental health services, and preventive programs aimed at adolescent populations. The adequate precision of the instrument at high levels of NSSI favors its usefulness as a screening tool for identifying adolescents with greater psychological vulnerability, emotional regulation difficulties, and potential suicidal risk (Wang et al., 2022; World Health Organization, 2025). In the professional field, having a brief, valid, and reliable instrument may facilitate psychological assessment processes, clinical monitoring, and timely referral of adolescents presenting indicators of emotional risk. Likewise, evidence of invariance across sex allows for valid comparisons between males and females, promoting psychological interventions that are more sensitive to the clinical and emotional differences reported between both groups (Moloney et al., 2024; Wang et al., 2023). In the Peruvian context, where significant gaps still exist in access to specialized adolescent mental health services and there is limited availability of culturally validated instruments for assessing NSSI, the ISAS-I could contribute to strengthening prevention programs, school screening protocols, and early interventions in educational institutions and primary mental health care centers (Baños-Chaparro, 2023; World Health Organization, 2025). Furthermore, its use could favor the generation of more precise epidemiological evidence on NSSI in Peruvian adolescents, contributing to the design of public policies aimed at promoting mental health and preventing suicidal risk in youth populations. Nevertheless, the ISAS-I total score should be interpreted with caution. The instrument provides a summary index of lifetime NSSI behavioral involvement, but it should not be understood as a direct indicator of clinical severity, medical risk, psychological function, or equivalence among different self-injurious behaviors. Therefore, its use as a screening tool should be complemented by item-level interpretation and, when appropriate, by a detailed clinical assessment of the specific behavior, recurrence pattern, medical risk, intent, psychological function, and contextual factors associated with self-injury.

Among the main strengths of the study are the large sample size and the incorporation of advanced psychometric analyses that had not previously been reported in ISAS-I research, particularly Bayesian reliability, measurement invariance, and item response theory (Bildik et al., 2013; Castro and Kirchner, 2017; Klonsky and Glenn, 2009; Pérez et al., 2021; Tian et al., 2025). These approaches allow for a more robust and precise evaluation of the psychometric properties of the instrument. However, the study also presents some limitations. First, the use of non-probabilistic sampling limits the generalizability of the results; therefore, future studies should incorporate probabilistic and representative samples from different regions of the country. Second, the cross-sectional design prevents the evaluation of temporal stability and causal relationships between variables; thus, future research could develop longitudinal studies to examine developmental changes in NSSI among adolescents. Likewise, the sample included greater participation from adolescents from rural areas of Huancavelica, which may introduce sociocultural biases in responses; consequently, future studies should include more heterogeneous samples from urban and rural contexts across different Peruvian regions.

Another limitation is that the present study did not conduct a full cross-cultural adaptation of the ISAS-I. Specifically, no forward and back translation, expert reconciliation, formal semantic or conceptual equivalence assessment, or cognitive interviewing was performed. The study used an existing Spanish adaptation and evaluated its content validity and comprehensibility in Peruvian adolescents through expert judgment and pilot administration. Therefore, the findings should be interpreted as psychometric evidence for the existing Spanish version in this population, rather than as evidence of a new cultural adaptation for Peru. Future studies should conduct a formal cross-cultural adaptation process, including cognitive interviews and detailed analyses of semantic, conceptual, and cultural equivalence.

In addition, the evidence based on expert judgment should be interpreted cautiously. The panel comprised four judges and the pilot administration involved seven adolescents, which may have limited the diversity of professional perspectives and the identification of potential comprehension problems. Therefore, these findings provide preliminary support for content validity and comprehensibility, but they do not replace a full cultural adaptation process or larger cognitive-interview studies. Additionally, the use of the PHQ-2 and GAD-2 as external criteria limited the breadth of the analysis because each instrument contains only two items and may not fully capture the complexity of depressive symptoms or generalized anxiety. Future studies should include a direct measure of NSSI or a clinical interview, as well as theoretically relevant variables such as emotion regulation, impulsivity, hopelessness, suicidal ideation, and adverse experiences, to strengthen validity evidence based on relationships with other variables.

Another important limitation concerns the assessment of suicidal intent. Although participants were instructed to report only self-injurious behaviors performed without suicidal intent, intent was not assessed separately for each ISAS-I method. This is particularly relevant for behaviors such as ingesting harmful substances, which may overlap with NSSI, suicide attempts, substance use, or other risky behaviors. Similarly, behaviors such as hair pulling and interfering with wound healing may overlap with body-focused repetitive behaviors. Therefore, the present findings should be interpreted as evidence based on self-reported NSSI according to the general ISAS-I instruction, rather than as the result of a clinical differentiation between NSSI, suicide attempts, behaviors of undetermined intent, or other repetitive behaviors. Future studies should include clinical interviews or item-specific probes to distinguish suicidal intent, medical risk, psychological function, and possible diagnostic overlap for each self-injurious method. Future longitudinal studies should examine test–retest reliability, longitudinal measurement invariance, and predictive validity for prospectively assessed NSSI-related outcomes. Where an appropriate clinical reference standard is available, diagnostic accuracy analyses (e.g., sensitivity and specificity) and differential item functioning analyses across sex and other relevant sociodemographic groups should also be conducted.

## Conclusion

The ISAS-I demonstrated adequate psychometric properties in Peruvian adolescents, including a unidimensional structure, adequate reliability, invariance across sex, consistent relationships with depressive symptoms and generalized anxiety, as well as favorable evidence based on IRT. These results support its usefulness as a valid and reliable instrument for the assessment of non-suicidal self-injury (NSSI) in Peruvian adolescents. However, the present study used an existing Spanish adaptation and evaluated its content validity, comprehensibility, and psychometric performance in the target population; therefore, the findings should not be interpreted as evidence of a new or complete cross-cultural adaptation for Peru. Furthermore, the study expands the international psychometric evidence of the ISAS-I through the incorporation of Bayesian analyses, measurement invariance, and item response theory, contributing evidence on the use of the existing Spanish adaptation in Peruvian adolescents.

## Data Availability

The raw data supporting the conclusions of this article will be made available by the authors, without undue reservation.
